# Semaphorin 6C Suppresses Proliferation of Pancreatic Cancer Cells via Inhibition of the AKT/GSK3/β-Catenin/Cyclin D1 Pathway

**DOI:** 10.3390/ijms23052608

**Published:** 2022-02-26

**Authors:** Yu-Hsuan Hung, Shih-Han Hsu, Ya-Chin Hou, Pei-Yi Chu, Yung-Yeh Su, Yan-Shen Shan, Wen-Chun Hung, Li-Tzong Chen

**Affiliations:** 1 National Institute of Cancer Research, National Health Research Institutes, Tainan 704, Taiwan; yhhung@nhri.edu.tw (Y.-H.H.); lucy75127@gmail.com (S.-H.H.); yysu@nhri.edu.tw (Y.-Y.S.); hung1228@nhri.edu.tw (W.-C.H.); 2 Institute of Clinical Medicine, College of Medicine, National Cheng Kung University, Tainan 704, Taiwan; yachi2016@yahoo.com.tw (Y.-C.H.); ysshan@mail.ncku.edu.tw (Y.-S.S.); 3 Department of Pathology, Show Chwan Memorial Hospital, Changhua 500, Taiwan; chu.peiyi@msa.hinet.net; 4 Division of General Surgery, Department of Surgery, National Cheng Kung University Hospital, Tainan 704, Taiwan; 5 Division of Hematology & Oncology, Department of Internal Medicine, College of Medicine, Kaohsiung Medical University, Kaohsiung 804, Taiwan; 6 Center for Cancer Research, Kaohsiung Medical University Hospital, Kaohsiung 807, Taiwan

**Keywords:** SEMA6C, CDK4/6, pancreatic cancer

## Abstract

Semaphorins (SEMAs) are axon guidance factors that participate in axonal connections and nerve system development. However, the functional roles of SEMAs in tumorigenesis are still largely uncovered. By using in silico data analysis, we found that SEMA6C was downregulated in pancreatic cancer, and its reduction was correlated with worse survival rates. RNA sequencing revealed that cell cycle-related genes, especially cyclin D1, were significantly altered after blockage of SEMA6C by neutralizing antibodies or ectopic expressions of SEMA6C. Mechanistic investigation demonstrated that SEMA6C acts as a tumor suppressor in pancreatic cancer by inhibiting the AKT/GSK3 signaling axis, resulting in a decrease in cyclin D1 expression and cellular proliferation. The enhancement of cyclin D1 expression and cyclin-dependent kinase activation in SEMA6C-low cancer created a druggable target of CDK4/6 inhibitors. We also elucidated the mechanism underlying SEMA6C downregulation in pancreatic cancer and demonstrated a novel regulatory role of miR-124-3p in suppressing SEMA6C. This study provides new insights of SEMA6C-mediated anti-cancer action and suggests the treatment of SEMA6C-downregulated cancer by CDK4/6 inhibitors.

## 1. Introduction

Semaphorins (SEMAs) are a family of proteins, originally identified as axon guidance factors, that play crucial roles in the maintenance of axonal connections and the migration of neuronal cells [1,2,3]. This axon guidance family is divided into five classes according to structural similarity, including secretory class 3, transmembrane classes 4–6, and membrane-anchored class 7. Among the transmembrane SEMAs, members in classes 4–5 are frequently reported to aid in cancer development, such as SEMA4A in the angiogenesis of breast cancer [4], SEMA4B in the proliferation of head and neck cancer [5], SEMA4C in colon cancer motility [6], and SEMA4D in the immune regulation of lung cancer [7]. SEMA5A has also been shown to modulate motility and metastasis of gastric and pancreatic cancers [8]. However, the importance of class 6 SEMAs in tumorigenesis is still largely unclear.

Previous studies have demonstrated that the expression of SEMA6A is associated with better survival in lung cancer and brain tumors [9,10,11], while the expression of SEMA6B is linked with increased incidence of thyroid tumors [12]. However, the underlying mechanisms by which class 6 SEMAs affect cancer cell behavior have seldom been addressed. SEMA6C was cloned as a novel class 6 SEMA and was named SEMAY at that time [13]. Subsequent investigations suggested a role of SEMA6C in controlling nerve growth cone distribution, spinal cord development, and skeletal muscle innervation [14,15,16].

Recently, SEMA6C has also been proposed to participate in the regulation of retinal development and ovarian function [17,18]; however, information on whether SEMA6C plays any role in the regulation of tumor growth has never been reported. In this study, we found that SEMA6C is a potential tumor suppressor in pancreatic cancer. Bioinformatics analysis and immunohistochemical staining showed the downregulation of SEMA6C in pancreatic tumor tissues. We revealed that SEMA6C suppressed cell proliferation via the AKT/GSK3/β-catenin/cyclin D1 axis, where the major associated partners of cyclin D1 that elicit its biological functions include cyclin-dependent kinase 4/6 (CDK4/6) [19]. Recent studies demonstrated that the CDK4/6 inhibitors exhibit potent growth-inhibitory activity in various cancers in vitro and in animal studies. Furthermore, a number of CDK4/6 inhibitors are under different phases of clinical trials [20,21]. Recently, several CDK4/6 inhibitors have been approved for the treatment of breast cancer in clinical settings [22]. We also tested the therapeutic effect of CDK4/6 inhibitors in SEMA6C-low pancreatic cancer, which exhibited increased cyclin D1 expression.

Furthermore, we explored the mechanism of SEMA6C downregulation in pancreatic cancer and found that miR-124-3p, a highly expressed oncogenic miRNA in pancreatic cancer, could suppress SEMA6C expression. Treatment of the miR-124-3p inhibitor induced an increase in SEMA6C expression and reduced the proliferation of pancreatic cancer cells. The anti-cancer function of SEMA6C revealed in the present study might provide additional clues for pancreatic cancer therapy.

## 2. Results

### 2.1. Semaphorin 6C Is Downregulated in Pancreatic Cancer and Is Associated with Reduced Patient Survival

**Figure 1:**
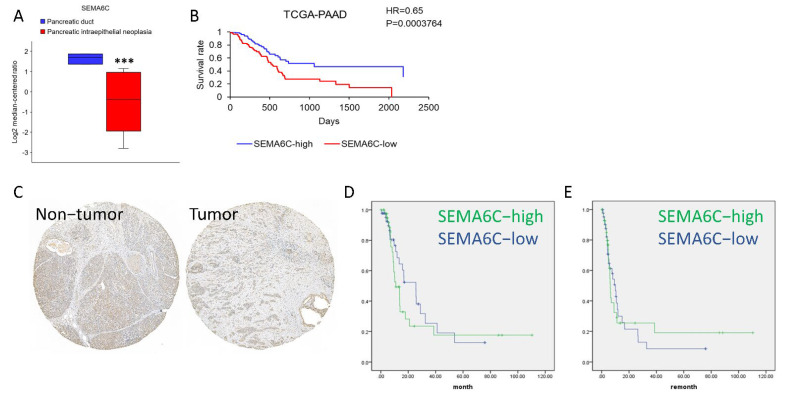
Relation between SEMA6C expression and pancreatic cancer formation. SEMA6C mRNA expression (**A**) and its association with survival (**B**) in pancreatic cancer datasets were analyzed. SEMA6C protein expression (**C**) in the tissue array mentioned above and its relation to overall survival (**D**) as well as disease-free survival (**E**) as shown. For Figure 1B, SEMA6C grouping was based on its median expression (SEMA6C-high n = 31; SEMA6C-low n = 56). For Figure 1D,E, SEMA6C grouping was also based on its mean expression (SEMA6C-high n = 42; SEMA6C-low n = 44). *** *p* < 0.001.

We first checked the expression of the members of the SEMA6 family in the Oncomine database and found a downregulation of SEMA6C in pancreatic cancer (Figure 1A). In addition, the survival rate of SEMA6C-low pancreatic cancer patients was significantly worse than that of SEMA6C-high patients (*p* < 0.001, Figure 1B). To validate the finding obtained from mRNA expression data, we examined SEMA6C protein levels in a set of pancreatic tumor tissues and found a decrease in SEMA6C in some pancreatic tumors (Figure 1C). Consistent with mRNA results, the overall survival and metastasis-free survival of the SEMA6C-low patients were worse (Figure 1D,E). These data suggested that SEMA6C could be a tumor suppressor gene in pancreatic cancer.

### 2.2. RNA Sequencing Reveals the Alteration of Cell Cycle Progression in SEMA6C-Low Pancreatic Cancer

**Figure 2:**
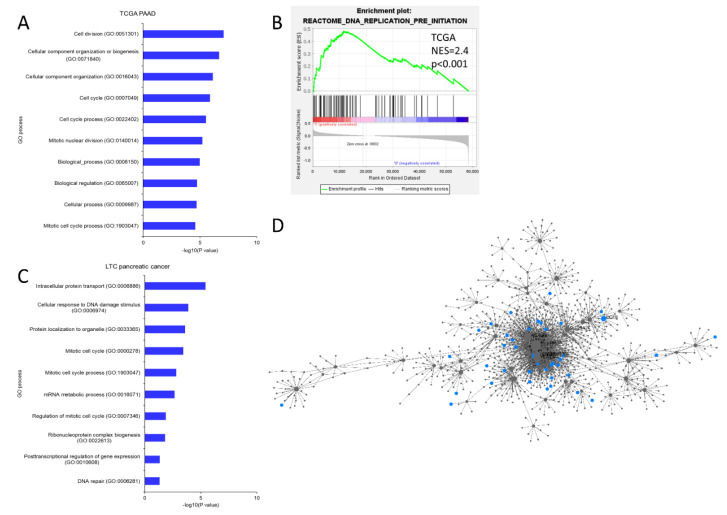
RNA sequencing reveals the alteration of cell cycle progression in SEMA6C-low pancreatic cancer. RNA sequencing results from TCGA (**A**,**B**) or the present study (**C**,**D**) were analyzed for SEMA6C-associated pathway (**A**,**C**), gene set (**B**), and network (**D**).

To characterize SEMA6C-low pancreatic cancer, we applied AmiGO to analyze the TCGA dataset for comprehensive survey and found enrichment in cell cycle dysregulation pathways, including cell division, cell cycle, and nuclear division (Figure 2A). Bioinformatics analyses using GSEA demonstrated the enrichment of gene signatures with DNA replication (Figure 2B). To validate these findings, we ectopically expressed SEMA6C in MIA PaCa-2 pancreatic cancer cells that had very low levels of endogenous SEMA6C expression and compared the expression profiles by using next-generation sequencing to identify the altered pathways. As shown in Figure 2C, several significantly changed pathways after SEMA6C overexpression were mitotic cell cycle, cellular response to DNA damage stimulus, and the regulation of cell cycles, consistent with the results of the TCGA dataset. In addition, NetworkAnalyst analysis of our RNA sequencing data revealed the enrichment of the cell proliferation-related network (Figure 2D). These results prompted us to study the effect of SEMA6C on the proliferation of pancreatic cancer cells.

### 2.3. SEMA6C Inhibited the Growth of Pancreatic Cancer Cells

**Figure 3:**
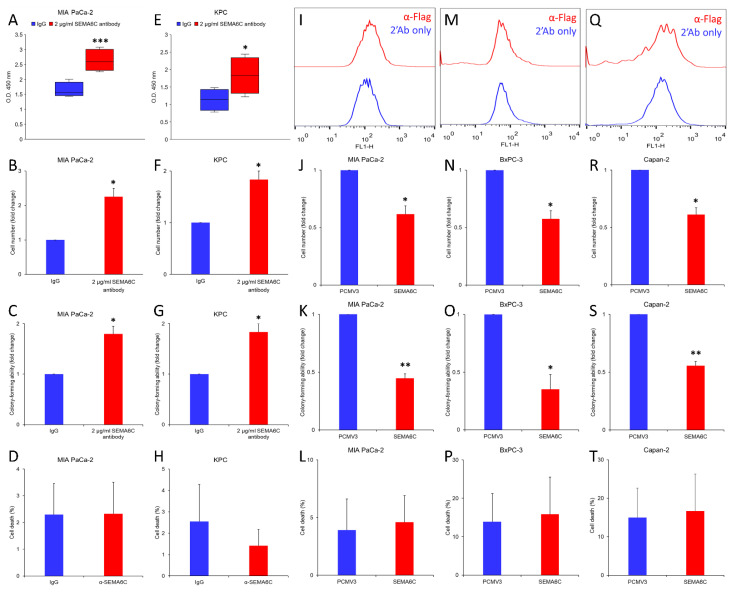
SEMA6C influences pancreatic cancer proliferation. Human pancreatic cancer cell lines MIA PaCa-2 (**A**–**D**, **I**–**L**), BxPC-3 (**M**–**P**), Capan-2 (**Q**–**T**) and mouse pancreatic cancer cell line KPC (**E**–**H**) were subjected to SEMA6C antibody-mediated blockage (**A**–**H**) or SEMA6C overexpression (**I**–**T**) and its effect on cell proliferation (**B**,**F**,**J**,**N**,**R**), colony formation (**C**,**G**,**K**,**O**,**S**) and apoptosis (**D**,**H**,**L**,**P**,**T**) was analyzed. * *p* < 0.05, ** *p* < 0.01, *** *p* < 0.001.

As SMEA6C is a transmembrane protein, we applied a neutralizing antibody to block its function. The antibody was specifically bound to the human pancreatic cancer cell line MIA PaCa-2 and the mouse pancreatic cancer cell line KPC (Figure 3A,E). Treatment with anti-SEMA6C antibodies enhanced the proliferation of pancreatic cancer cells, consistent with the anti-cancer function of SEMA6C in this cancer (Figure 3B,F). Additionally, the transforming activities assessed by colony formation also dramatically increased (Figure 3C,G), although anti-SEMA6C antibodies did not affect cell viability (Figure 3D,H). To verify our conclusion, we selected additional human pancreatic cancer cell lines including BxPC-3 (KRAS wildtype, TP53 mutant) and Capan-2 (KRAS mutant, TP53 mutant, RNF43 mutant) for an SEMA6C overexpression study. Flow cytometry analysis confirmed the increase in cell surface SEMA6C in MIA PaCa-2, BxPC-3, and Capan-2 cells (Figure 3I,M,Q). A reduction in cell proliferation was observed after SEMA6C expression, consistent with its tumor-suppressive role (Figure 3J,N,R). Moreover, colony-forming activity was also inhibited (Figure 3K,O,S), although SEMA6C overexpression did not affect cell death (Figure 3L,P,T). These results suggest that SEMA6C suppressed the proliferation of pancreatic cancer cells without promoting cell death.

### 2.4. SEMA6C Affects Cell Proliferation via the AKT/GSK3/β-Catenin/Cyclin D1 Axis

**Figure 4:**
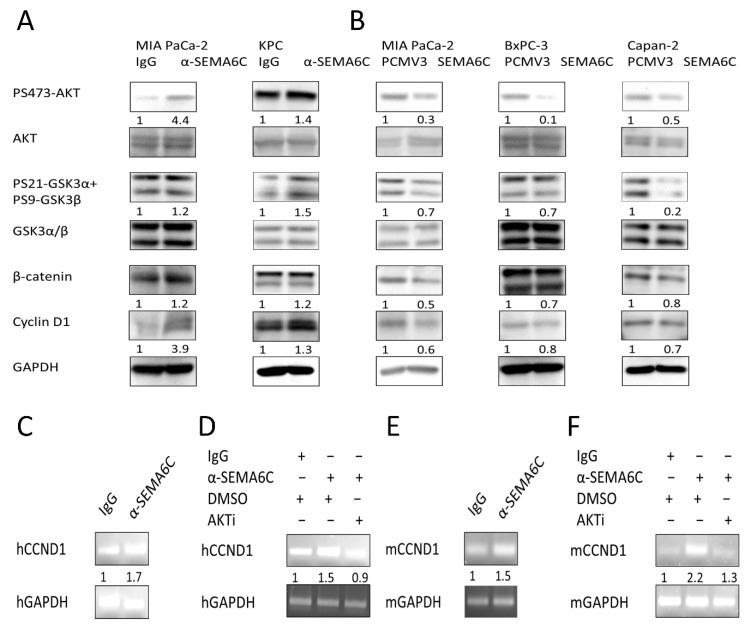
SEMA6C affects cell proliferation via AKT/GSK3/β-catenin/cyclin D1 axis. Human pancreatic cancer cell lines MIA PaCa-2, BxPC-3, and Capan-2 and mouse pancreatic cancer cell line KPC were subjected to SEMA6C antibody-mediated blockage or SEMA6C overexpression, and its effect on target expressions at protein level (**A**,**B**) or mRNA level (**C**–**F**) was shown.

Previous studies have demonstrated that SEMA6C inhibits AKT activation in mouse ovary cells [18,23]. We investigated the involvement of AKT in the SEMA6C-inhibited growth of pancreatic cancer cells. SEMA6C blockage by using neutralizing antibodies increased AKT phosphorylation in human MIA PaCa-2 and mouse KPC cells (Figure 4A). Phosphorylation of GSK3, a downstream target of AKT, was also enhanced, resulting in increased β-catenin expression (Figure 4A). In contrast, SEMA6C overexpression in three pancreatic cancer cells suppressed AKT/GSK3 activity and β-catenin expression (Figure 4B). Cyclin D1 is a transcriptional target for β-catenin to control cell cycle progression [24]. Consistently, the cyclin D1 protein was upregulated upon SEMA6C blockage, while it was decreased after SEMA6C overexpression. Our data also showed the increment of cyclin D1 mRNA by SEMA6C neutralizing antibodies in MIA PaCa-2 and KPC cells, suggesting that SEMA6C blockage enhanced cyclin D1 via transcriptional activation (Figure 4C,E). The inhibition of AKT activity by a specific inhibitor reversed the upregulation of cyclin D1 by SEMA6C antibody treatment (Figure 4D,F). These results suggest that SEMA6C modulates cyclin D1 expression and proliferation via the AKT/GSK3/β-catenin axis in pancreatic cancer cells.

### 2.5. CDK4/6 Inhibitor Palbociclib Suppresses Proliferation in SEMA6C-Low Pancreatic Cancer

**Figure 5:**
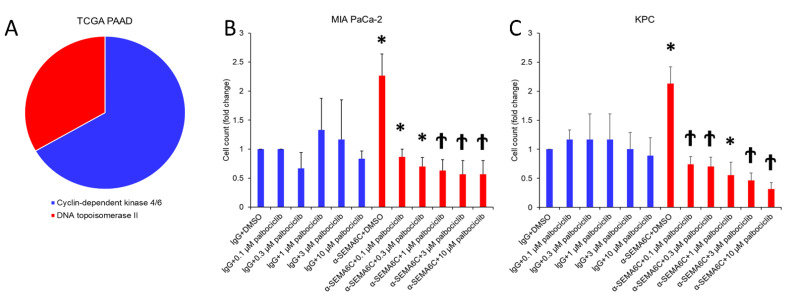
CDK4/6 inhibitor Palbociclib suppresses proliferation in SEMA6C-low pancreatic cancer. TCGA-based and L1000CDS^2^-predicted SEMA6C-mimicking therapeutics are shown (**A**) and CDK4/6 inhibitor was selected for in vitro validation of proliferation in MIA PaCa-2 (**B**) and KPC (**C**). * represents *p* < 0.05; † represents *p* < 0.01.

To search for therapeutic targets in pancreatic cancer with SEMA6C downregulation, we applied L1000CDS^2^ analysis and found that cell cycle-related inhibitors, including those against topoisomerase or CDK4/6, may be potential drugs for the treatment of SEMA6C-low pancreatic tumors (Figure 5A). The identification of CDK4/6 inhibitors is not surprising, because we have demonstrated the upregulation of cyclin D1, the major cyclin complexes with CDK4 and CDK6, after SEMA6C blockage. Our data showed that the CDK4/6 inhibitor, Palbociclib, indeed decreased the proliferation of MIA PaCa-2 and KPC cells in a dose-dependent manner (Figure 5B,C), suggesting the therapeutic potential of a CDK4/6 inhibitor for SEMA6C-low pancreatic cancer.

### 2.6. miR-124-3p Inhibitor Suppresses Pancreatic Cancer Proliferation via SEMA6C Upregulation

**Figure 6:**
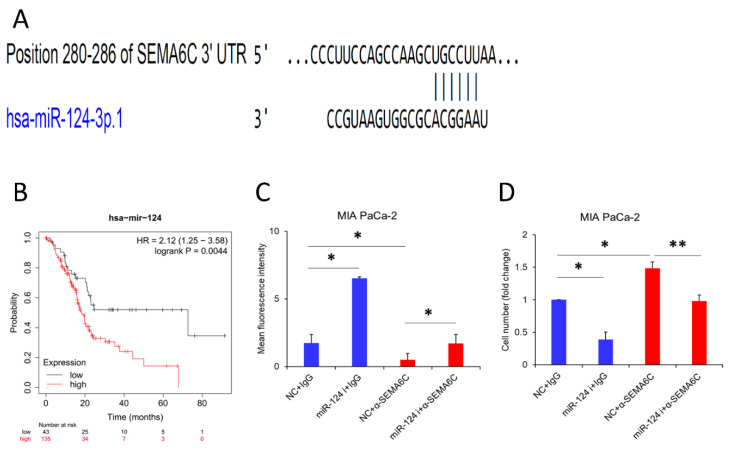
The miR-124-3p inhibitor suppresses pancreatic cancer proliferation via SEMA6C upregulation. miRNAs targeting SEMA6C were identified by overlapping predicted miRNAs from miRDB, TargetScan, and miRWalk. The targeting of SEMA6C by miR-124-3p (**A**) and the prognosis prediction by this miRNA in pancreatic cancer (**B**) are shown. The effect of miR-124-3p inhibitors on SEMA6C expression (**C**) and proliferation (**D**) in MIA PaCa-2 was analyzed. * represents *p* < 0.05, ** represents *p* < 0.01.

We next addressed the mechanism of SEMA6C downregulation in pancreatic tumors. Post-transcriptional regulation of SEMA6C by miRNA was considered. By bioinformatics prediction using miRDB, TargetScan, and miRWalk, miR-124-3p was found to be a common SEMA6C-targeting miRNA. This miRNA complexes perfectly with the position 280–286 of 3′UTR of SEMA6C mRNA (Figure 6A). In addition, high miR-124-3p expression was associated with poor prognosis in pancreatic cancer (Figure 6B). For mechanistic study, we focused on MIA PaCa-2 cells, which expressed high levels of miR-124-3p (Supplementary Figure S1). Treatment of miR-124-3p inhibitors restored SEMA6C expression and inhibited the proliferation of MIA PaCa-2 cells (Figure 6C,D), while the increase in cellular growth by SEMA6C antibody blockage was also reversed by miR-124-3p inhibitors. These results suggest that miR-124-3p is a critical upstream regulator of SEMA6C in pancreatic cancer cells.

## 3. Discussion

Recently, the potential roles of SEMA genes in neoplasia development have been reviewed [9,25,26]. However, the functions of SEMAs in tumorigenesis are complex and are tumor type-specific. For example, SEMA5A was found to be upregulated in pancreatic cancer and was correlated with tumor growth, invasion, and metastasis [27]. However, the secreted form of SEMA5A suppressed the proliferation of pancreatic cancer cells [28] while also displaying tumor-promoting activities in gastric cancer [29]. Conversely, this SEMA was suggested to act as a tumor suppressor in lung cancer [30]. Among the members of the SEMA6 family, SEMA6B and SEMA6D have been implicated in cancer formation. SEMA6B promotes the progression of glioma via activating its cognate receptor plexin A4 [31]. Similarly, SEMA6D is increased in gastric cancer and its expression is linked with tumor angiogenesis and metastasis [32,33]. However, the expression and function of SEMA6C are currently unclear.

In the present study, we demonstrated that SEMA6C suppressed the proliferation of human and mouse pancreatic cancer cells. Additionally, we revealed that AKT/GSK3 signaling is the major downstream pathway inhibited by SEMA6C to elicit its anti-cancer activity. In SEMA6C-downregulated pancreatic cancer, the enhancement of AKT activity induced the accumulation of nuclear β-catenin to promote the expression of cyclin D1 and stimulated cell growth. The mammalian cell cycle is controlled by different cyclin-CDK complexes, which could phosphorylate the retinoblastoma protein to relieve its inhibitory action on DNA replication and cell division [34]. In the past decades, the development of CDK inhibitors has been an active area in cancer research. Pan-CDK inhibitors targeting various CDKs showed potent anti-cancer activity; however, they also exhibited significant adverse effects in patients. Thus, selective CDK inhibitors are more suitable for clinical applications. The progression of the cell cycle in the early G1 phase is mainly regulated by cyclin D1/CDK4/6.

Our finding that SEMA6C-low pancreatic cancer showed increased cyclin D1 expression motivated us to test whether CDK4/6 inhibitors may have therapeutic benefits on pancreatic cancer with the downregulation of SEMA6C. Indeed, Palbociclib, also known as PD 0332991, a clinically approved CDK4/6 inhibitor, suppresses SEMA6C-low cancer cells more effectively, suggesting a druggable target in these cancers. By performing L1000CDS^2^ analysis, we also identified topoisomerase inhibitors as potential drugs for the treatment of SEMA6C-low pancreatic cancer. Recently, a topoisomerase I inhibitor, pegylated liposomal irinotecan, has been approved for use in patients with metastatic pancreatic ductal adenocarcinoma [35]. The selection of patients by using SEMA6C as a marker may lead to more precise cancer therapy.

Another important finding of this study is the elucidation of the mechanism of SEMA6C downregulation in pancreatic cancer. MiRNAs are short noncoding RNAs that may modulate the expression of genes via translational inhibition or mRNA proteolysis [36]. The involvement of miRNAs in pancreatic cancer has been well documented recently [37]. For example, anti-cancer miR-203 directly targets SEMA6A to trigger apoptotic cell death in oral cancer cells [38]. In addition, miR-506 and miR-23a-3p have been shown to suppress SEMA6D in osteosarcoma and colon cancer, respectively [39,40]. Our identification of SEMA6C targeting by miR-124-3p demonstrates a novel regulatory miRNA in pancreatic cancer. Moreover, we also found that a high expression of miR-124-3p is linked with worse survival rates in pancreatic cancer patients.

The SEMA6C-staining analysis of tissue array showed a tendency for better survival rates in SEMA6C-high patients. The results did not reach statistical significance due to the limited case number. While SEMA6C-low pancreatic cancer patients had lower survival rates at the end point of the analyses, the difference was minor. We then searched for possible reasons. If we focused on paired normal–cancerous tissues, the downregulation of SEMA6C at the protein level during pancreatic tumorigenesis is more obvious (n = 6 pairs, Supplementary Figure S2). This suggests that high SEMA6C expression is still a good predictor for better prognosis in pancreatic cancer patients. In addition, the SEMA6C protein is always downregulated during pancreatic tumorigenesis. Therefore, we concluded that SEMA6C plays a tumor-suppressive role in pancreatic cancer.

Collectively, this study provides new insights as to how SEMA6C modulates the proliferation of pancreatic cancer cells.

## 4. Materials and Methods

### 4.1. Oncomine and PROGgene Analyses

Oncomine (https://www.oncomine.org/resource/login.html) (last accessed on 21 September 2018) and PROGgene (http://www.compbio.iupui.edu/proggene) (last accessed on 21 September 2018) analyses were performed to investigate the expression alteration and prognosis prediction by SEMAs in 20 cancer types. The default setting was applied, and the result was collected before October 2018.

### 4.2. cBioPortal, AmiGO, Geneset Enrichment Analysis (GSEA), and Network Analyst Analyses

cBioPortal (https://www.cbioportal.org/) [41] (last accessed on 21 September 2018) analyses were performed to identify expression patterns, prognostic prediction, clinical association, and coexpressed gene signature for SEMA6C in pancreatic cancer. Briefly, a TCGA pancreatic cancer dataset was analyzed for SEMA6C coexpressed genes, which were then ranked according to Spearman’s correlation. The top 500 positively or negatively correlated genes were extracted as SEMA6C coexpressed gene signatures, then AmiGO, GSEA, and NetworkAnalyst analyses were performed to identify enriched pathways in SEMA6C-low pancreatic cancer.

### 4.3. RNA Sequencing

A sequencing library was constructed with a TruSeq Stranded mRNA Library Prep Kit (Illumina; San Diego, CA, USA) following the manufacturer’s instructions. mRNA was purified from 1 μg RNA by oligo(dT)-coupled magnetic beads and first-strand cDNA was synthesized using random primers and reverse transcriptase. After the generation of double-strand cDNA and adenylation on 3′ ends of DNA fragments, adaptors were ligated and purified with the AMPure XP system (Beckman Coulter; Brea, CA, USA). The quality of the libraries was assessed on Bioanalyzer 2100 (Agilent; Santa Clara, CA, USA) and real-time PCR. Qualified libraries were sequenced using NovaSeq 6000 (Illumina) with 150 bp paired-end reads generated by Genomics (New Taipei, Taiwan). Filtered reads were aligned to reference genomes with Bowtie2 (version 2.3.4.1). Transcript quantification was performed with RSEM (version 1.2.28). The identification of differentially expressed genes was performed with EBSeq (version 1.16.0).

### 4.4. L1000CDS^2^ Analysis

L1000CDS^2^ (https://maayanlab.cloud/l1000cds2/#/index) [42] (last accessed on 21 September 2018) was applied to identify counteracting drugs for SEMA6C-low pancreatic cancer from TCGA, and the predicted drugs were presented as frequency.

### 4.5. Cell Culture and Treatment

Human pancreatic cancer lines MIA PaCa-2 (BCRC; Hsinchu, Taiwan) and Capan-2 (ATCC; Manassas, VA, USA) were cultured in DMEM (HyClone; Logan, UT, USA) with 10% fetal calf serum (FBS) and 1X penicillin-streptomycin (HyClone), as described previously [43,44]. Human pancreatic cancer cell line BxPC-3 (BCRC; Hsinchu, Taiwan) and mouse pancreatic cancer cell line KPC (provided by Dr. Kuang-Hung Cheng from Institute of Biomedical Sciences, National Sun Yat-sen University, Kaohsiung, Taiwan) were cultured in RPMI (HyClone) with the same supplements. For antibody treatment, cells were treated with 2 μg/mL rabbit control IgG (10201, LEADGENE; Tainan, Taiwan) or SEMA6C antibody (GTX118489, GeneTex; Hsinchu, Taiwan) for 24 h. For drug treatment, cancer cells were treated with CDK4/6 inhibitor Palbociclib (Sigma; Burlington, MA, USA) at the indicated concentration for 24 h, and the cells were harvested for analysis. For plasmid transfection, cells were transfected with 2 μg PCMV3 vector control (CV020, Sino Biological; Beijing, China) or SEMA6C-overexpressing plasmid (HG23441-NF, Sino Biological), together with 3 μL HyFect (LEADGENE) for 48 h. For miRNA transfection, 200 nM control miRNA inhibitors or miR-124-3p inhibitors (GenePharma; Shanghai, China) were applied, and the cancer cells were collected 48 h after transfection.

### 4.6. Antibody Internalization Assay

Antibody internalization assay was performed as described previously [45,46] with minor modifications. Briefly, cells at the concentration of 5000 per ml were seeded onto 96-well plates. After 24 h, control IgG or SEMA6C antibody was added. One hour post-antibody addition, cells were washed with PBS, fixed with 4% paraformaldehyde (PFA) for 10 min, and permeabilized with 0.05% Tween 20 PBS for 10 min. An internalized antibody was detected by a polymer-conjugated secondary antibody (NICHIREI, Tokyo, Japan) and then by 3, 3′, 5, 5′-tetramethylbenzidine (TMB; Sigma). The reaction was stopped by 2N H_2_SO_4_, and the end product was measured at O.D. 450 nm by FlexStation 3 (Molecular Probes; Waltham, MA, USA).

### 4.7. Flow Cytometry

Cells were fixed with 4% PFA for 10 min, stained with primary antibody in 1% BSA-PBS for 1 h, and then stained with a secondary antibody for another 1 h. Stained cells were subjected to analyses with FASCalibur’s flow cytometer, and the data were analyzed by FlowJo software (BD; Franklin Lakes, NJ, USA).

### 4.8. Proliferation and Colony Formation Assays

Cell proliferation was analyzed by a trypan blue exclusion test, and colony formation was performed as described previously [47]; briefly, 10,000 cells of indicated condition were seeded onto 6-well plates and cultured in the wells for seven days. Formed colonies were fixed with methanol, stained with methyl blue, and destained with ddH_2_O. Colonies were counted under a microscope (IX71, OLYMPUS; Tokyo, Japan).

### 4.9. Cell Death Assay

Cell death was analyzed with propidium iodide uptake at a concentration of 50 μg/mL at the end of treatment. Stained dead cells were counted under a microscope.

### 4.10. Immunofluorescence

Cells were fixed with 4% PFA for 10 min and stained with a primary antibody in 1% BSA-PBS overnight at 4 °C. Cells were incubated with a fluorescent secondary antibody for 1 h and 5 μg/mL DAPI (Sigma) for 5 min. Signals were detected with a fluorescent microscope (IX71, OLYMPUS) and analyzed with ImageJ software.

### 4.11. Western Blotting

Cells were lyzed in CelLytic M (Sigma) with 1 mM Na_3_VO_4_, 5 μg/mL aprotinin and 1 mM phenylmethanesulfonylfluoride (PMSF) for 20 min at 4 °C. After centrifugation at 12,000 rpm for 15 min at 4 °C, supernatants were harvested and protein concentrations were quantified with Bradford reagent (Bio-Rad; Hercules, CA, USA). Equal amounts of protein from each sample were mixed with 5× sample dye (0.1% bromophenol blue, 8% dithiothreitol, 10% SDS, 50% glycerol, and 250 mM Tris-HCl pH 6.8), heated at 95 °C for 5 min, and subjected to gel electrophoresis. After transfer at 100V for 1.5 h, polyvinylidene difluoride membranes were blocked with 5% BSA for 1 h and then incubated with the primary antibody. After incubation with the secondary antibody, the membranes were washed extensively, and the signals were detected by ECL (PerkinElmer; Waltham, MA, USA) and analyzed with UVP (Analytik Jena; Jena, Germany). The results were quantified with ImageJ software. Antibodies used in this study included those against phospho-AKT (Ser473) (9271, Cell Signaling; Danvers, MA, USA), AKT (GTX121937, GeneTex), phospho-GSK3α/β (Ser21/9) (8566, Cell Signaling), GSK3α/β (5676, Cell Signaling), β-catenin (9587, Cell Signaling), cyclin D1 (GTX108624, GeneTex), and GAPDH (GTX627408, GeneTex).

### 4.12. RNA Extraction and Reverse Transcription-Polymerase Chain Reaction (RT-PCR)

Cells were lyzed in Trizol (Invitrogen; Waltham, MA, USA), and RNA was extracted according to manufacturer’s instructions. RNA concentration was determined by a nanophotometer (Implen; Munich, Germany), and 2 μg RNA was reversely transcribed into cDNA using ReverTra Ace set (PURIGO; Taipei, Taiwan) according to manufacturer’s instructions. PCR was performed with MyCycler (Bio-Rad) under the conditions of 98 °C for 20 s, 65 °C for 1 min, and 72 °C for 1 min for a total of 35 cycles. The PCR product was subjected to electrophoresis with 1% agarose gel (VWR; Radnor, PA, USA) under 100 V for 15 min and stained with 7500×-diluted EtB“Out” (Sigma) for 20 min. The signals were analyzed with AlphaImager (Alpha Innotech, San Leandro, CA, USA) and ImageJ software. The PCR primer sequences were as follows: hCCND1-F, GCTGCGAAGTGGAAACCATC; hCCND1-R, CCTCCTTCTGCACACATTTGAA; hGAPDH-F, AGAAGGCTGGGGCTCATTTG; hGAPDH-R, AGGGGCCATCCAC AGTCTTC; mCCND1-F, CTCCGTATCTTACTTCAAGTGCG; mCCND1-R, CTTCTCGGCAGTCAAGGGAA; mGAPDH-F, AGGTCGGTGTGAACGGATTTG; mGAPDH-R, TGTAGACCATGTAGTTGAGGTCA.

### 4.13. miRNA and KM Plotter Analyses

SEMA6C-targeting miRNAs were identified from miRDB (http://mirdb.org/) [48] (last accessed on 9 November 2020), TargetScan (http://www.targetscan.org/vert_72/) [49] (last accessed on 9 November 2020) and miRWalk (http://mirwalk.umm.uni-heidelberg.de/) [50] (last accessed on 9 November 2020), and the miRNAs overlapping in the three databases were selected for further analysis. The common miRNA, miR-124-3p, was tested for prognosis prediction in pancreatic cancer using a KM plotter (https://kmplot.com/analysis/) [51] (last accessed on 9 November 2020).

### 4.14. Tissue Array and Immunohistochemistry Staining

Specimens from pancreatic cancer patients that had undergone surgical resection were approved by the Institutional Review Board of National Cheng Kung University Hospital (A-ER-105-459) and the National Health Research Institute (EC1090507-E). Clinical information between 2001 and 2012 was reviewed using electronic medical records. Tissue arrays were constructed from formalin-fixed paraffin-embedded blocks of 86 specimens, as described previously [52]. After deparaffinizing and rinsing with 10 mM Tris-HCl (pH 7.4) and 150 mM sodium chloride, slides were treated with methanol and 3% H_2_O_2_, and then heated at 100 °C for 20 min in 10 mM citrate buffer. The slides were incubated with the SEMA6C antibody for 1 h and washed with PBS, and the signals were developed using EnVision Detection Systems, Peroxidase/DAB, Rabbit/Mouse kit (Dako; Santa Clara, CA, USA). The slides were analyzed under a microscope and SEMA6C expression was evaluated by an experienced pathologist, with scoring defined by staining intensity and the percentage of positive cells expressed as follows: 0, no expression; 1, weak expression; 2, moderate expression; and 3, strong expression.

### 4.15. Statistical Analysis

Statistical analysis was performed with SPSS Statistics 17.0 (IBM; Armonk, NY, USA). Statistical differences between control and experimental groups were calculated with Student’s *t*-test, and *p* < 0.05 was considered statistically significant (* represents *p* < 0.05; ** and † represent *p* < 0.01; *** represents *p* < 0.001).

## Data Availability

The data that support the findings of this study are available from the corresponding author upon reasonable request.

## Supplementary Materials

The following supporting information can be downloaded at: https://www.mdpi.com/article/10.3390/ijms23052608/s1.
