# Supplementary material for: Semaphorin 6C Suppresses Proliferation of Pancreatic Cancer Cells via Inhibition of the AKT/GSK3/β-Catenin/Cyclin D1 Pathway
Source: Int J Mol Sci. 2022 Feb 26;23(5):2608. doi: 10.3390/ijms23052608 (PMC8910270; doi:10.3390/ijms23052608)
Supplement: Supplementary file 1 [file ijms-23-02608-s001.zip › ijms-1604941-supplementary.pdf]

Supplementary Figures

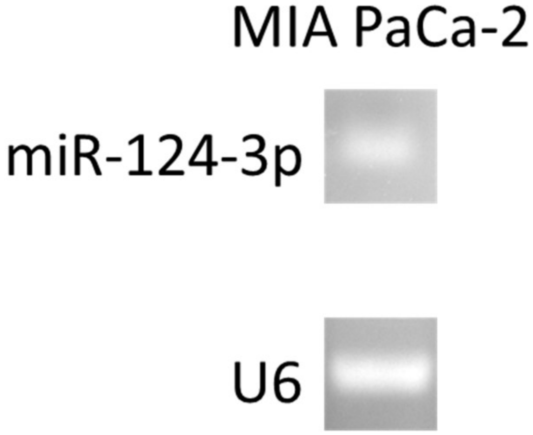

Supplementary Figure S1. miR-124-3p expression in MIA PaCa-2 cells. The expression of miR-124-3p and internal control U6 was analyzed with RNA from MIA PaCa-2 cells, which was reversely transcribed and amplified by specific primers mentioned in Supplementary Method. PCR product was analyzed with electrophoresis.

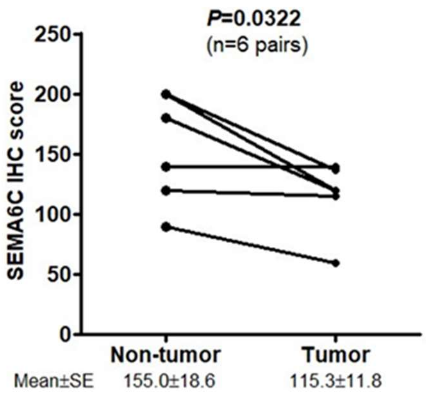

Supplementary Figure S2. SEMA6C expression in paired normal-cancerous pancreatic tissues. SEMA6C expression in paired normal-cancerous tissues (n=6 pairs) in tissue array was analyzed.

Supplementary Method

Reverse transcription and polymerase chain reaction for miRNA

2 μg RNA was reversely transcribed (PURIGO, Taipei, Taiwan) into cDNA with specific miRNA RT primers or random primer for internal control U6 spliceosome small RNA (Plant Methods. 2007;3:12.). PCR was performed as 98°C, 20 seconds; 60°C, 15 seconds; 72°C, 1 minute for 40 cycles. For internal control the annealing temperature was 65°C and cycle number was 35. PCR product was analyzed with electrophoresis on 2.5% TBE-agarose gel under 100 V for 30 minutes and stained with 2 μg/ml ethidium bromide (Bio-Rad, Hercules, CA, USA) for 10 minutes. Primer sequences were designed as in reference (Plant Methods. 2007;3:12.) and listed as below: miR-124-3p-RT, GTCGTATCCAGTGCAGGGTCCGAGGTATTCGCACTGGATACGACTTGG CA; miR-124-3p-PCR-F, GTATACTAAGGCACGCGGTGA; miR-124-3p-PCR-R, GTGCAGGGTCCGAGGT; U6-PCR-F, GCTTCGGCAGCACATATACTAAAAT; U6-PCR-R, CGCTTCACGAATTTGCGTGTCAT.
